# Acute effects of MDMA on trust, cooperative behaviour and empathy: A double-blind, placebo-controlled experiment

**DOI:** 10.1177/0269881120926673

**Published:** 2020-06-15

**Authors:** Anna Borissova, Bart Ferguson, Matthew B Wall, Celia JA Morgan, Robin L Carhart-Harris, Mark Bolstridge, Michael AP Bloomfield, Tim M Williams, Amanda Feilding, Kevin Murphy, Robin J Tyacke, David Erritzoe, Lorna Stewart, Kim Wolff, David Nutt, H Valerie Curran, Will Lawn

**Affiliations:** 1 Clinical Psychopharmacology Unit, UCL, London, UK; 2 NIHR University College London Hospitals Biomedical Research Centre, University College Hospital, London, UK; 3 UMC Utrecht Brain Center, University Medical Center Utrecht, Utrecht, The Netherlands; 4 Invicro London, London, UK; 5 Neuropsychopharmacology Unit, Centre for Psychiatry, Imperial College London, London, UK; 6 Psychopharmacology and Addiction Research Centre, University of Exeter, Exeter, UK; 7 Centre for Psychedelic Research, Department of Psychiatry, Imperial College London, London, UK; 8 Translational Psychiatry Research Group, Research Department of Mental Health Neuroscience, Division of Psychiatry University College London, London, UK; 9 The Traumatic Stress Clinic, St Pancras Hospital, Camden and Islington NHS Foundation Trust, London, UK; 10 National Hospital for Neurology and Neurosurgery, London, UK; 11 Beckley Foundation, Oxford, UK; 12 Cardiff University Brain Research Imaging Centre, Cardiff, UK; 13 School of Biomedical Sciences, King’s College London, London, UK

**Keywords:** MDMA, empathy, prosocial, trust, cooperative behaviour

## Abstract

**Background:**

3,4-Methylenedioxymethamphetamine (MDMA) is being actively researched as an adjunct to psychotherapy. It may be beneficial to trust, empathy and cooperative behaviour due to its acute prosocial effects.

**Aim:**

To test (a) the acute effects of MDMA on measures of empathy, trust and cooperative behaviour, and (b) subacute changes in mood three days after MDMA administration.

**Methods:**

Twenty-five participants (*n*=7 female), participated in this double-blind, repeated-measures, placebo-controlled experiment. Participants attended two acute sessions, one week apart. Each acute session was followed by a subacute session three days later. Participants received placebo (100 mg ascorbic acid) during one acute session, and MDMA (100 mg MDMA-HCl) at the other, with order counterbalanced. Participants completed the following tasks assessing prosocial behaviour: a trust investment task, a trustworthy face rating task, an empathic stories task, a public project game, a dictator game and an ultimatum game. Participants reported subjective effects. Blood was taken pre-drug, 2 and 4 hours post-drug, and tested for plasma MDMA levels.

**Results:**

MDMA acutely increased self-reported ‘closeness to others’ and ‘euphoria’ and increased plasma concentrations of MDMA. MDMA did not significantly change task-based empathy, trust or cooperative behaviour. Using Bayesian analyses, we found evidence that MDMA and placebo did not differ in their effects on empathy and cooperative behaviour. MDMA did not significantly change subacute mood and this was supported by our Bayesian analyses.

**Conclusion:**

Despite augmentation in plasma MDMA levels and subjective drug effects, we found no increase in prosocial behaviour in a laboratory setting.

## Introduction

3,4-Methylenedioxymethamphetamine (MDMA) is a drug being actively researched as a possible adjunct to psychotherapy, with 17 Phase II clinical trials either ongoing or already completed (Schenberg, 2018). Phase II trials focusing on patients with posttraumatic stress disorder (PTSD) have yielded large (0.8) effect sizes (Mithoefer et al., 2019). The mechanism by which MDMA might improve psychotherapy outcomes is yet to be established. Testing MDMA’s effect on social processes and behaviours in laboratory studies has yielded mixed results (Kamilar-Britt and Bedi, 2015).

Acutely, MDMA consistently induces self-reported prosocial effects when given in clinical trials and when used recreationally (‘recreational MDMA’). For recreational users, these have been reported as an increased feeling of ‘closeness’ to others and greater interactions with others (Baylen and Rosenberg, 2006). In laboratory studies, the subjective effects of MDMA include feelings of ‘sociable’ or ‘gregarious’ (Bedi et al., 2009; Kirkpatrick et al., 2014a; Kirkpatrick et al., 2014b; Kirkpatrick et al., 2014c), ‘close to others’ (Hysek et al., 2012; Hysek and Liechti, 2012; Hysek et al., 2014; Schmid et al., 2014), and ‘trusting’ (Dolder et al., 2018; Schmid et al., 2014). However, laboratory evidence concerning MDMA’s prosocial effects on trust, empathy and cooperative behaviour is mixed.

Emotional empathy appears to be enhanced by MDMA (Kuypers et al., 2014; Hysek et al., 2014; Schmid et al., 2014). For cognitive empathy, MDMA may impair accurate recognition of negative emotions (Dolder et al., 2018; Hysek et al., 2012; Hysek et al., 2014; Wardle and de Wit, 2014; Bedi et al., 2010). However, null drug effects on cognitive empathy have also been reported (Hysek et al., 2014; Schmid et al., 2014; Gabay et al., 2019; Kuypers et al., 2014).

Results are mixed for the effect of MDMA on perceived trustworthiness. A naturalistic study, in which participants took their own recreational MDMA and were assessed in their homes, reported increases (Stewart et al., 2014). Conversely, one laboratory study found a null effect (Kirkpatrick et al., 2014c).

In the same naturalistic study, Stewart and colleagues also assessed the effect of recreational MDMA on cooperative behaviour, finding a prosocial effect on both the ultimatum and dictator games. A laboratory experiment of MDMA on the ultimatum game also found a prosocial effect (Gabay et al., 2018).

Other laboratory studies have found positive effects of MDMA on the social value orientation (SVO) (Hysek et al., 2014), the prisoner’s dilemma (Gabay et al., 2019) and the welfare-trade off tasks (Kirkpatrick et al., 2015). At a lower (75 mg) dose, others have found null effects of MDMA on a trust game, which assesses trust and cooperative behaviour (Kuypers et al., 2014), and on the SVO (Schmid et al., 2014). In summary, existing research into MDMA’s effects on empathy, trust and cooperative behaviour has produced inconsistent results.

Use of recreational MDMA has been shown to lead to low mood a few days after having taken the drug: the ‘mid-week blues’ (Curran and Travill, 1997). This has also been found in a laboratory study of MDMA 24 hours after drug administration (Liechti et al., 2001), though this was not replicated in a pooled analysis of nine other studies (Vizeli and Liechti, 2017). Low mood in the 7 days after administration has also been noted in some clinical trials of MDMA (Ot’alora G et al., 2018). Given some inconsistencies, it remains important to further characterise the subacute effects of MDMA on mood. This is particularly relevant given interest in MDMA as an adjunct to psychotherapy.

We aimed firstly to extend and replicate the findings from a naturalistic study of recreational MDMA’s acute prosocial effects (Stewart et al., 2014) in a controlled laboratory experiment, using measures of empathy, trust and cooperative behaviour. Secondly, we aimed to assess subacute changes to mood three days after MDMA administration. We hypothesised that relative to placebo: (a) MDMA would increase prosocial behaviour on assessments of cooperative behaviour, trust ratings and empathy and (b) MDMA would lead to subacute low mood.

## Materials and methods

### Design

Participants were enrolled in a within-subject, double-blind, placebo-controlled study with drug order balanced across participants. Participants attended four sessions in total, with two acute drug administration sessions and two subacute sessions. The acute drug administration sessions were 7 days apart. The subacute sessions occurred 3 days after each of the acute sessions. Data collection was conducted between January and October 2012.

The study was approved by National Research Ethics Service (NRES) West London Research Ethics Committee, Imperial College London’s Joint Compliance and Research Office, Imperial College Healthcare NHS Trust and Imperial College London’s Faculty of Medicine, University College London Research Ethics Committee, and was conducted in accordance with Good Clinical Practice guidelines. A Home Office Licence was obtained for the storage and handling of a Schedule 1 drug and Imperial College London sponsored the research. Participants gave informed consent.

### Participants

Participants were 25 healthy, right-handed, poly-drug (including MDMA) users, aged 18 and 58 years. Participants were required to abstain from MDMA for 7 days and other psychoactive drugs for at least 48 hours. This was confirmed by a urine screen and self-reported drug use. Participants took an alcohol breathalyser test to confirm that they had no recent alcohol consumption.

Participants underwent a screen of their general health and present mental health. Inclusion criteria were: (a) currently mentally and physically healthy as determined by a psychiatric interview and medical screen and (b) at least one previous experience with MDMA. For details of medical screening and additional exclusion criteria see Supplementary Materials. Five of the participants were videoed as part of a UK television documentary (Channel 4©) on the effects of MDMA.

### Assessments

#### Empathic stories task:

This was a novel task. It measured emotional empathy by assessing participants’ emotional reactions in response to stories with different emotional themes. There were two ‘happy’ themed stories, two ‘angry’ themed stories, and two ‘sad’ themed stories. Participants were asked to rate how ‘good’ to ‘bad’ the stories made them feel on a scale of 1 (most positive) to 9 (most negative).

##### Trustworthy face rating task:

This task followed the procedure of Stewart et al. (2014), based on Winston et al. (2002) . Participants were shown 33 male and 33 female emotionally neutral faces taken from the Karolinska Directed Emotional Faces database (Lundqvist and Litton, 1998). Participants were asked to rate how trustworthy the faces were on a scale of 1–7.

#### Cooperative behaviour games

##### Public project game (Ledyard, 1994):

Participants were asked how much of £5 they would like to contribute to the public project. All contributions would then be added together, multiplied by two and distributed equally among everyone who took part in the study (not just those who had contributed).

##### Dictator game (Hoffman et al., 1996):

Participants were told they had been given £5 and were asked to choose how to split this amount with another person in the study.

##### Ultimatum game (Thaler, 1988; Guth and Tietz, 1990):

***Proposer role*:** Participants were asked to decide how to split £5 with another person in the study.

***Decider role*:** Participants were told that the proposer participant had decided how they would split £5 with them. They were asked to write down the minimum offer they would be willing to accept. Participants were told that if the decider participant accepted the offer, both parties would receive the amounts agreed; if rejected, both parties would receive nothing.

##### Trust investment task:

This task followed the procedure of Berg et al. (1995). Briefly, participants were told they had £500, which they could choose to invest in 20 different entrepreneurs. They were shown the face of the individual running the business and asked to choose an amount that they wished to invest. Participants were told they would be paid a proportion of the money they won.

Every participant completed every task in the order above. For full details on all the tasks see the Supplementary Materials.

### Self-rated assessments

#### Mood and symptom visual analogue scales

We report 11 Acute visual analogue scales (VASs). Participants rated how they were feeling at that moment on a 0–10 scale, anchored by two opposing statements. The scales reported here are: no euphoria – extreme euphoria, no drug effect – strong drug effect, no jaw clenching – severe jaw clenching, lethargic – energy, trusting of others – distrusting of others, no empathy – extreme empathy, friendly – hostile, no feelings of closeness to others – strong feelings of closeness to others, amicable – antagonistic, want to be alone – want to be with others, compassionate – indifferent. The scales combined the scales in Stewart et al. (2014) and additional scales based on previous MDMA studies (Hoshi et al., 2004).

We report five subacute VASs. The scales reported here are: happy – sad, calm – anxious, trusting of others – distrusting of others, want to be alone – want to be with others, no empathy – extreme empathy.

##### Beck Depression Inventory (Beck et al., 1996):

Participants completed the 21-item Beck Depression Inventory (BDI). On acute sessions, participants answered for how they have felt over the previous 2 weeks, and on the subacute sessions participants answered for how they have felt over the previous 3 days (Curran and Travill, 1997). Higher scores reflect higher depression severity.

##### State-Trait Anxiety Inventory (Spielberger, 1970):

This questionnaire has 20 items for assessing trait anxiety and 20 items for assessing state anxiety. All items are rated on a scale of 1 (almost never) – 4 (almost always). Higher scores reflect greater anxiety.

#### MDMA plasma concentration

MDMA was determined by using liquid chromatography – mass spectrometry by ViaPath, King’s College Hospital. MDMA concentrations were only analysed on the MDMA condition.

#### Drug and dosing parameters

Participants were administered 100 mg encapsulated MDMA-HCl orally and on a separate occasion, placebo (100 mg encapsulated ascorbic acid).

#### Procedure

Participants first attended a screening visit in which they completed a baseline BDI and State-Trait Anxiety Inventory (STAI) questionnaire and had general medical and psychiatric assessments. They then completed the acute sessions and subacute sessions.

The acute sessions involved participants completing task-based measures of trust, empathy and cooperative behaviour and self-report assessments of mood. Participants also completed an MRI scan, results for which have been reported elsewhere (Carhart-Harris et al., 2015; Carhart-Harris et al., 2014).

Tasks reported in this paper began 2 hours after drug administration and were completed 4 hours after drug administration. STAI questions were measured pre-drug at the first acute visit.

**Figure 1.**
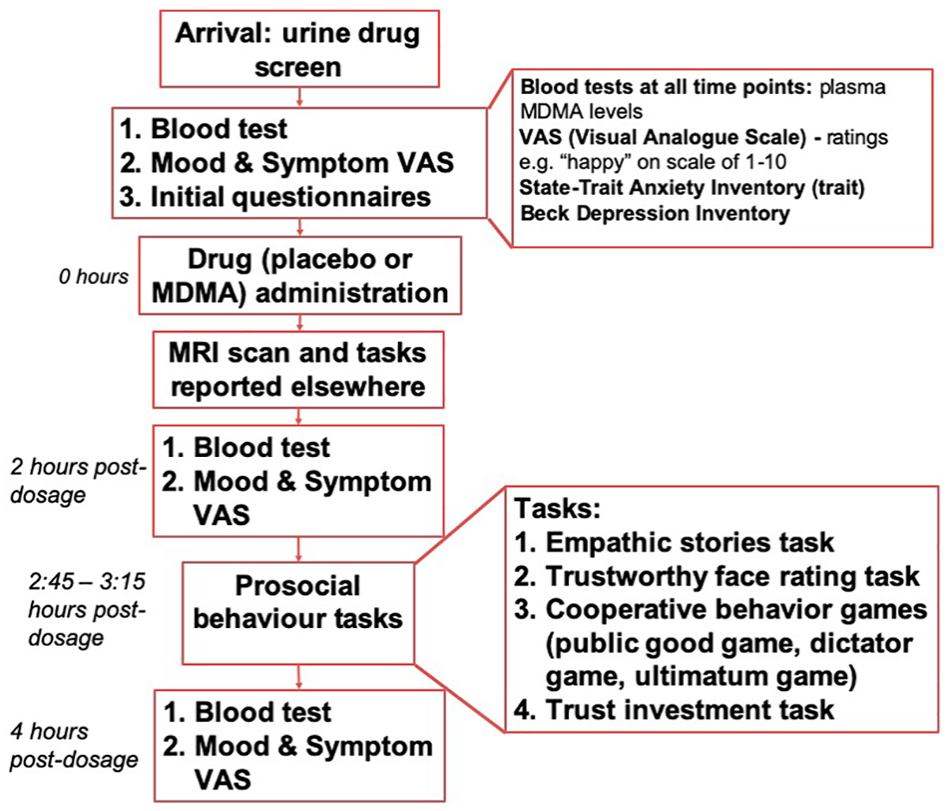
Flowchart to represent the order of events on each acute session day.

See Figure 1 for a representation of an acute session day.

On subacute sessions participants completed five mood and symptom VASs. They also completed a modified BDI. Participants did not complete any tasks on subacute sessions.

## Statistical analyses

All data were analysed using IBM Statistical Package for Social Sciences (IBM SPSS version 26). Our analyses reported here include all participants (filmed and not-filmed). Excluding participants who were filmed did not affect the significance or direction of the results so they were kept in the analyses.

### MDMA plasma concentrations analyses

MDMA plasma concentrations were analysed with repeated measures analysis of variance (RM-ANOVA) with time as the within-subject factor.

### Acute VAS

Eleven mood and symptoms VASs were analysed with RM-ANOVA with Drug and Time as within-subject factors. We Bonferroni-corrected the α-level to 0.0045 to account for multiple comparisons due to the 11 VASs.

### Subacute VAS/BDI

Five subacute mood and symptom VASs were analysed with RM-ANOVA with drug and day as within-subject factors. We compared pre-drug VAS ratings at acute sessions with subacute session ratings. We Bonferroni-corrected the α-level to 0.01 to account for multiple comparisons due to the five VASs.

BDI ratings were analysed with RM-ANOVA with drug and day as within-subject factors. We compared pre-drug BDI ratings at acute sessions with subacute BDI ratings.

### Task data

Normality of task data and statistical assumptions were assessed and appropriate tests were employed.

The empathic stories task was analysed using RM-ANOVA. The dependent variable was the rating score of 1–9. Drug and story emotion (happy, angry, sad) were within-subjects factors. Trustworthy face rating task data were analysed using RM-ANOVA. The dependent variable was the mean trust rating. Drug and face gender were within-subject factors. Trust investment task data were analysed with paired two-tailed t-tests, to compare MDMA and the placebo. The dependent variable was the total amount invested. Cooperative behaviour tasks data were analysed with bootstrapped bias-corrected and accelerated (BCa) confidence-interval paired two-tailed t-tests, because data were nonparametrically distributed. For the public project game, dictator game and ultimatum game proposer role the dependent variables were the amount of money donated by the participant. For the ultimatum game decider role the dependent variable was the amount written as the minimum amount accepted by the participant.

Drug order was added as an additional between-subjects factor and results were compared with reported primary analyses (without drug order). Results were unaffected by drug order, unless otherwise noted. For all analyses, significant main effects and interactions were followed up by Bonferroni-corrected post hoc comparisons using the inbuilt function in SPSS syntax. *P* values were considered statistically significant at <0.05, unless otherwise stated.

We also calculated Jeffreys–Zellner–Siow (JZS) Bayes factors using an online calculator (http://pcl.missouri.edu/bayesfactor) to evaluate evidence in favour of the null hypotheses for t-tests. We used a scaled-information prior of *r* = 1, which is the default value recommended (Rouder et al, 2009). We used a cut-off of JZS Bayes Factor greater than 3 as evidence for the null hypothesis (Rouder et al., 2009)

#### Correlation analyses:

We tested the association between peak (2 hour post-drug) MDMA and task variables, and the mood and symptom VASs which showed an effect of MDMA. We set the α-level to 0.005 for task variables, and 0.01 for the mood and symptom VASs to account for multiple comparisons. We also tested the association between behavioural and subjective responses. We tested the correlation of trust task responses with the ‘trusting of others’ VAS and empathy task responses with the ‘empathy’ VAS. We set the α-level to 0.008. We calculated Pearson’s *r*, with bootstrapped confidence intervals where data were not normally distributed.

## Results

### Demographics

We tested 7 women and 18 men with a mean age of 34.1 (SD=10.6, range 21–58) years. Participants had a mean BDI score of 2.19 (SD=2.7, range= 0–8), corresponding to no depression, and mean trait STAI scores of 34.6 (SD=7.6, range= 21-46), corresponding to low/no anxiety.

All participants reported at least one previous use of MDMA, with a median 10 lifetime uses (interquartile range=3-45, range 1–200). Other lifetime drug use reported by participants can be found in the Supplementary Materials.

### MDMA plasma concentration (Figure 2)

MDMA plasma concentrations increased from 0 to a mean of 213.8777±14.10ng/ml and 211.249±18.505ng/ml, two and four hours after MDMA treatment, respectively. There was a main effect of time (F_2,42_=115.541, *p*<0.001, *η_p_*^2^=0.846), reflecting an increase from pre-drug to two hours post-administration (t_21_=15.169, *p*<0.001, mean difference=213.877, 95% CI: 177.199 to 250.555, *d*=3.234) and to four hours post-administration (t_21_=11.416, *p*<0.001, mean difference=211.249, 95% CI: 163.111 to 259.387, *d*=2.434).

**Figure 2.**
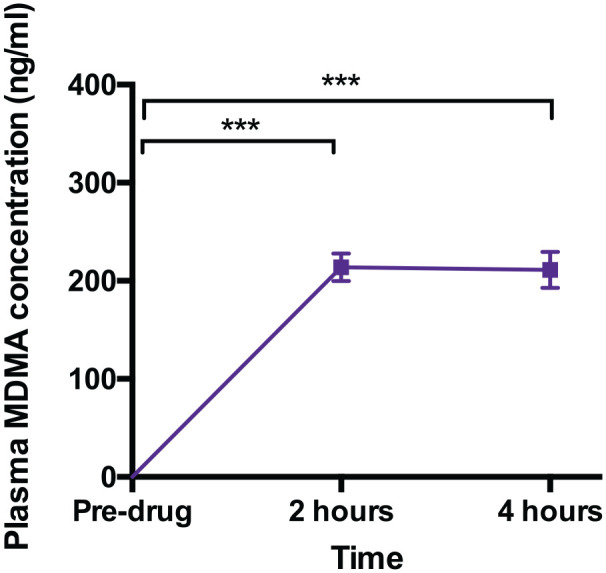
Plasma MDMA concentrations pre- and post-MDMA. (***= *p*<0.001)

### Subjective effects

#### Acute effects (Figure 3)

**Figure 3.**
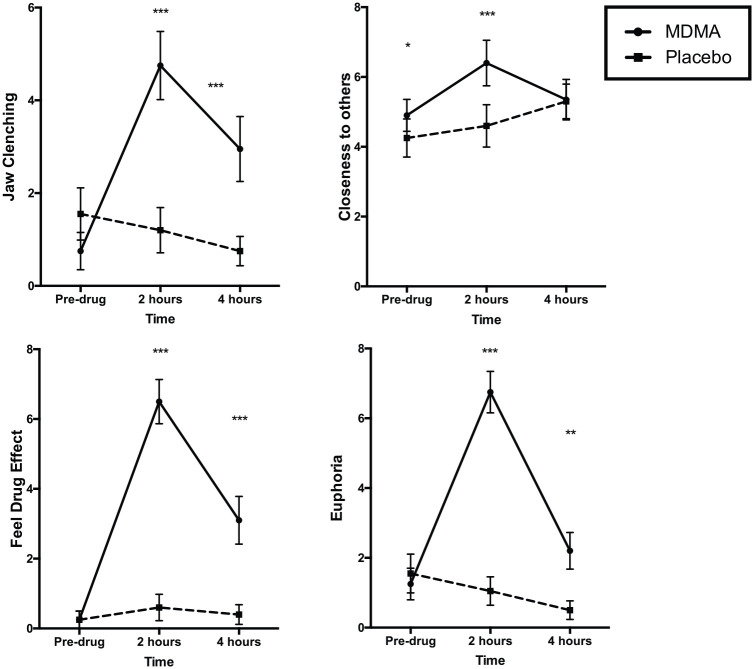
Subjective effects of MDMA compared to Placebo at 0, 2 and 4 hours post-drug. The dashed lines represent the Placebo condition. *** = *p*⩽0.001; **=*p*<0.005, *=*p*<0.05. These denote the comparison between MDMA and placebo ratings at each time point.

##### Feel drug effect:

There was an interaction between drug and time for overall ‘feel drug effect’ (F_2,38_=43.125, *p*<0.001, *η_p_*^2^=0.694). MDMA increased ‘feel drug effect’ ratings from baseline at both the 2 hour (*p*<0.001) and 4 hour (*p*=0.001) time points post-administration, while ratings on placebo were not significantly changed (*ps*=0.780-0.990). At 2 hours and 4 hours post-drug, MDMA-induced ‘feel drug effect’ ratings were significantly greater than placebo (two hours: *p*<0.001, four hours: *p*=0.008).

##### Euphoria:

There was a drug by time interaction for ‘euphoria’ ratings (F_2,38_=44.519, *p*<0.001, *η_p_*^2^=0.701). MDMA increased ‘euphoria’ ratings at 2 hours post-drug relative to baseline (*p*<0.001), while placebo did not (*p*=0.745). Euphoria ratings were significantly greater at 2 and 4 hours post-MDMA compared to placebo (2 hours: *p*<0.001, 4 hours: *p*=0.003)

##### Jaw clenching:

There was a significant drug by time interaction for jaw clenching (F_2,38_=14.812, *p*<0.001, η_p_^2^=0.694), reflecting higher ratings at 2 hours and 4 hours post-drug for MDMA compared with placebo (*p*<0.001).

##### Closeness to others:

There was a drug by time interaction for closeness to others ratings (F_2,38_=8.010, *p*=0.001, *η_p_*^2^=0.297). Ratings increased post-MDMA at 2 hours relative to baseline (*p*=0.025), with no increase post-placebo (*p*=1.000). Ratings were significantly higher when comparing MDMA to placebo at baseline (*p*=0.039) and at 2 hours (*p*=0.002). There was a trend main effect of drug (F_1,19_=6.013, *p*=0.024, η_p_^2^=0.240) and time (F_2,38_=3.719, *p*=0.033, η_p_^2^=0.164).

We also found trend main effects of drug for empathy (F_1,18_=10.073, *p*=0.005, η_p_^2^=0.359) and ‘compassionate’ (F_1,18_=8.041, *p*=0.011, η_p_^2^ =0.309).

There were no other significant main effects or interactions of MDMA on ratings of energetic, trusting of others, empathy, friendliness, wanting to be with others, compassion or amicability to others. For full statistical results see Supplementary Materials table S3.

#### Subacute effects

There was a significant main effect of day for ratings of anxious (F_1,16_=11.506, *p*=0.004, η_p_^2^ =0.418). This reflected a decrease in anxiety ratings from baseline to day 3 (*p*=0.004), across both drug conditions.

There were no significant main effects of drug and day nor an interaction between drug and day on self-rated scales of happy, trusting of others, want to be with others and empathy. Bayesian analysis showed that ratings on all these scales were unchanged pre-MDMA and 3 days post-MDMA.

There were no significant main effects of drug and day nor an interaction between drug and day on BDI scores. Bayesian analysis provided evidence that BDI scores were unchanged pre-MDMA and 3 days post-MDMA (JZS Bayes Factor=3.096). For full results see Supplementary Materials, Table S4.

### Task Results

#### Empathic stories task

Data were missing for four participants, so we analysed 21 participants’ data. There was a significant main effect of story emotion (F_1.395, 27.907_=85.842, *p*<0.001, *η_p_*^2^=0.811), but no main effect of drug (F_1,20_=1.900, *p*=0.183, *η_p_*^2^ =0.087) or interaction (F_2,40_=0.932, p=0.402, *η_p_*^2^ =0.045). This reflected that ‘happy’ stories led participants to feel more positive than ‘angry’ stories (*p*<0.001), and ‘sad’ stories led to more negative feelings than both ‘happy’ (*p*<0.001) and ‘angry’ stories (*p*<0.001). Furthermore, Bayesian analysis provided evidence in favour of the null hypothesis that MDMA had no effect, compared to placebo, on ratings. The null hypothesis was almost six times more likely than the alternative hypothesis for rating of ‘happy’ and ‘sad’ stories. For full results see Supplementary Materials, Table S2.

#### Trustworthy face rating task

Data were missing for one participant so we analysed 24 participants’ data. There was no significant interaction effect between drug and face gender (F_1,23_=1.191, *p*=0.286, η_p_^2^ =0.049), or main effects of drug (F_1,23_=1.826, *p*=0.190, η_p_^2^ =0.074) or face gender (F_1,23_=0.790, *p*=0.383, η_p_^2^ =0.033). Furthermore, Bayesian analysis provided evidence in favour of the null hypothesis, showing that MDMA had no effect on ratings of perceived trustworthiness. The null hypothesis was almost six times more likely than the alternative hypothesis for rating of male faces. For full results see Supplementary Materials, Table S2.

#### Trust investment task

Data were missing for two participants, and two participants did not complete the task on the placebo condition so we analysed data for 21 participants. The amount of money invested did not differ between placebo and MDMA (t_20_=-1.636, *p*=0.117, mean difference=-683.905, 95% CI: -1555.892 to 188.082, *d*=0.357). Bayesian analysis did not provide evidence for either hypothesis. We found a significant interaction between drug and order (F_1,19_=11.923, *p*=0.003, η_p_^2^=0.065). This is explored in the Supplementary Materials, Table S2.

#### Cooperative behaviour games

##### Public project game:

Data were missing for three participants so we analysed 22 participants’ data. There was no significant difference between the MDMA (mean=4.727, SD=0.767) and placebo (mean=4.807, SD=0.681) conditions in amounts donated (t_*21*_=-0.675, *p*=0.503, mean difference= -0.080, BCa 95% CI: -0.318 to 0.125, *d*=0.144). Bayesian analysis yielded scaled JZS Bayes factor=4.924, indicating that the null hypothesis was almost five times more likely than the alternative hypothesis.

##### Dictator game:

Data were missing for three participants so we analysed 22 participants’ data. There was no significant difference between the MDMA (mean=2.818, SD=1.900) and placebo (mean=2.773, SD=1.932) conditions in amounts donated by participants (t_*21*_ = 0.211, *p*=0.848, mean difference = 0.045, BCa 95% CI: -0.341 to 0.500, *d*=0.045). Bayesian analysis yielded scaled JZS Bayes factor=5.995, indicating that the null hypothesis was almost six times more likely than the alternative hypothesis.

##### Ultimatum game: Proposer:

Data were missing for four participants so we analysed 21 participants’ data. There was no significant difference between the MDMA (mean=3.477, SD=1.198) and placebo (mean=3.124, SD=1.242) conditions (t_*20*_=1.124, *p*=0.294, mean difference=0.353, BCa 95% CI: -0.265 to 1.067, *d*=0.246). Bayesian analysis yielded scaled JZS Bayes factor=3.329, indicating that the null hypothesis was three times more likely than the alternative hypothesis.

##### Decider:

Data were missing for four participants so we analysed 21 participants’ data. There was no significant difference between the MDMA (mean=1.738, SD=0.983) and placebo (mean=1.715, SD=0.844) conditions, (t_*20*_= 0.127, *p*=0.918, mean difference=0.023, BCa 95% CI: -0.357 to 0.362, *d*=0.027). Bayesian analysis yielded scaled JZS Bayes factor=5.949, indicating that the null hypothesis was almost six times more likely than the alternative hypothesis.

### Correlations

There were no significant correlations between plasma MDMA concentration, task performance and mood and symptom VASs. Ratings on the VAS trusting of others and empathy did not significantly correlate with trust or empathy measured by task. See Supplementary Materials for full results.

## Discussion

We examined the prosocial effects of MDMA in a laboratory using a repeated-measures, double-blind, placebo-controlled experiment. We investigated social processes believed to be mechanistically important for MDMA’s benefit as an adjunct to psychotherapy: trust, cooperative behaviour and empathy. MDMA significantly increased subjective measures of euphoria, feel drug effects, jaw clenching and closeness to others. However, we found no significant effects of MDMA on task-based measures of prosocial behaviour and Bayesian analyses largely supported the null hypotheses that there were no differences between MDMA and placebo.

There are extensive reports that MDMA enhances self-reported subjective trust (Schmid et al., 2014; Dolder et al., 2018). This augmentation in trust is thought to be important for the value of MDMA as an adjunct to psychotherapy (Sessa, 2017). However, in laboratory settings, when using task-based measures of trust, null effects of MDMA are often reported (Kuypers et al., 2014; Kirkpatrick et al., 2014c). We found that MDMA did not increase subjective ratings of feeling ‘trusting of others’; participants were not more trusting of others with their money. We also found support for this null finding (that MDMA did not affect perceived trustworthiness of others’ faces or cooperative behaviour) through the Bayesian analysis. Subjectively-rated trust did not correlate with trust measured by tasks, which suggests these may involve different psychological processes.

We found no evidence that MDMA affects emotional empathy (though it did increase scores of closeness to others). This contrasts with some previous work, which has found that MDMA selectively affects emotional empathy (Hysek et al., 2014; Kuypers et al., 2014; Schmid et al., 2014). However, this effect has not always been consistently reported and may depend on a participants’ gender (Hysek et al., 2014) or occur only for positively valenced stimuli (Schmid et al., 2014).

We found a null effect on the cooperative behaviour games and the trustworthy face rating task. This may seem surprising, as these tasks have been found to be sensitive to recreational MDMA in a naturalistic setting (Stewart et al., 2014). However, the between-subject design with non-blinded participants in this previous study may have contributed to an expectation of drug effects. Additionally, the lack of information about the dose or purity of the recreational MDMA used complicates interpretation of this earlier study. It may also be that the controlled laboratory setting, as opposed to testing within participants’ homes, dampened the prosocial effects. The importance of context when administering a psychoactive drug has been recognised particularly with psychedelic treatment (Carhart-Harris et al., 2018). Indeed, a comfortable physical setting is recommended as a key part of MDMA-assisted psychotherapy (Mithoefer, 2015).

Some differences between the tasks in our study and previous ones yield important insights. In the ultimatum game, we asked participants to respond with the minimum offer they would accept. This – as opposed to the ‘direct-response’ method used by Gabay et al. (2018) where participants are presented with different offers to accept or reject – can lead to less punishment of unfair offers. Indeed, our participants were willing to accept offers that were below 40% of the total stake – below what is considered to be a ‘fair’ offer (Gabay et al., 2014). We also used a ‘one-shot’ task for our cooperative behaviour games. This contrasts to Gabay et al. (2018) and Gabay et al. (2019), where participants received feedback on their performance over multiple rounds. Such feedback might be important to more closely match real-world social interaction and allow for detection of an effect of MDMA. This again suggests there may be an important role for context, whereby true social feedback may be necessary to elicit effects of this drug – but this would need to be tested experimentally.

In the context of previous mixed findings, a possible interpretation of our results is that MDMA may not enhance all aspects of prosocial behaviour. We add to existing evidence that in a laboratory setting, perceived trustworthiness and financial trust do not seem affected by MDMA. Perhaps MDMA may augment specific, rather than all, prosocial behaviours. Additionally, as suggested by some of the factors in previous studies (sex differences, different effects for positive versus negative stimuli), these specific effects may not be seen in all individuals in all situations. Future studies will need to further focus on which specific aspects of prosocial behaviour are affected by MDMA and address whether these are moderated by sex, dose, top-up doses and positive versus negative stimuli. It would be useful to test whether MDMA affects participants’ willingness to trust others with their personal feelings or confidential information differently to financial tasks (such as with the envelope task paradigm of Mikolajczak et al. (2010)) and whether physical setting impacts on MDMA effects.

More generally, tasks which are completed on paper or via a computer are inherently different to the interpersonal process of psychotherapy. In order to delineate MDMA’s prosocial effects in relation to the psychotherapeutic process, it may be necessary to use procedures which involve measurements of interpersonal behaviour and to utilise frameworks for measuring the effects of context as proposed in Carhart-Harris et al. (2018). For instance, it would be valuable to extend the work of Baggott et al. (2016) on the effect of MDMA on more intimate sharing by assessing differences in outcomes in a comfortable versus neutral setting, with patient groups, and with varying levels of responsiveness of the researcher listening to the memory.

Corroborating results from some laboratory reported adverse effects (Vizeli and Liechti, 2017), and contrasting results from recreational MDMA use (Curran and Travill, 1997; Stewart et al., 2014) we did not find increased depression in the subacute visits (i.e. there were no ‘mid-week blues’). This was supported by Bayesian analysis. This phenomenon may have been attributable to lack of sleep and/or interactions with other recreational drugs used (Sessa, 2017). This is clearly important to know when planning future clinical trials of MDMA; it does not necessarily produce low mood when administered at these doses in safe settings.

### Strengths and limitations

A key strength of our acute study is that it was a placebo-controlled, double-blind experiment. For schedule I drugs, these studies are difficult, expensive and time-intensive to conduct. Our experiment therefore partially addresses the issue of participant and experimenter expectation, present in the previous naturalistic study (Stewart et al., 2014). Our analysis of MDMA in plasma demonstrated that the drug was successfully absorbed to levels similar to other studies (Kuypers et al., 2014; Vizeli and Liechti, 2018). We examined prosocial feelings and behaviour across a wide range of self-report and task-based assessments, for a comprehensive investigation of MDMA’s effects. Our results were consistently null with support from Bayesian analyses across our task-based assessments of prosocial behaviour.

However, we must acknowledge limitations. Our participants experienced pronounced subjective effects, which will have contributed to unblinding. Whilst the dose used lies in the range typically used for this type of research (e.g. within 75 mg–125 mg), in the psychotherapeutic setting higher doses and top-up doses midway through the session are often used. It is possible that a higher dose would have resulted in more pronounced MDMA effects on our tasks. It is also possible that some tasks were conducted post-peak effect, however, MDMA prosocial subjective and task effects have been reported 4 hours post administration (Vizeli and Liechti, 2018; Hysek et al., 2014). Our participants still significantly felt the effects of the drug and had MDMA in their plasma an hour after these tasks were completed.

## Conclusion

In conclusion, in a controlled laboratory setting, MDMA did not have an effect on measures of prosocial behaviour, despite increases in self-report levels of closeness to others, feel drug effect and euphoria. In the future, research should test the effects of MDMA on more ecologically valid measures with more focus on the context, as both could be important for the effects of MDMA. This will allow delineation of which prosocial effects of MDMA are moderated by setting, and therefore could aid the development of MDMA-assisted psychotherapy.

## Supplemental Material

MDMA_Prosocial_Effects_Supplementary_revision_submission – Supplemental material for Acute effects of MDMA on trust, cooperative behaviour and empathy: A double-blind, placebo-controlled experiment Click here for additional data file. Supplemental material, MDMA_Prosocial_Effects_Supplementary_revision_submission for Acute effects of MDMA on trust, cooperative behaviour and empathy: A double-blind, placebo-controlled experiment by Anna Borissova, Bart Ferguson, Matthew B Wall, Celia JA Morgan, Robin L Carhart-Harris, Mark Bolstridge, Michael AP Bloomfield, Tim M Williams, Amanda Feilding, Kevin Murphy, Robin J Tyacke, David Erritzoe, Lorna Stewart, Kim Wolff, David Nutt, H Valerie Curran and Will Lawn in Journal of Psychopharmacology
