# Supplementary material for: Acute effects of MDMA on trust, cooperative behaviour and empathy: A double-blind, placebo-controlled experiment
Source: J Psychopharmacol. 2020 Jun 15;35(5):547–55. doi: 10.1177/0269881120926673 (PMC8155732; doi:10.1177/0269881120926673)
Supplement: MDMA_Prosocial_Effects_Supplementary_revision_submission – Supplemental material for Acute effects of MDMA on trust, cooperative behaviour and empathy: A double-blind, placebo-controlled experiment [file MDMA_Prosocial_Effects_Supplementary_revision_submission.pdf]

Acute effects of MDMA on trust, cooperative behaviour and empathy: a double-blind, placebo-controlled experiment

A. BORISSOVA[1,10], B. FERGUSON[2], M.B. WALL[1,3,4], C. MORGAN[13], R.L. CARHART-HARRIS[4,12], M. BOLSTRIDGE[4], M.A.P. BLOOMFIELD[1,8,9,10,11], T.M. WILLIAMS[4], A. FEILDING[5], K. MURPHY[6], R.J. TYACKE[4], D. ERRITZOE[4,12], L. STEWART[1], K. WOLFF[7], D. NUTT[4], H.V. CURRAN[1], W. LAWN[1].

Supplementary Materials

Methods

Participants

Medical screening involved routine blood tests, electrocardiogram, heart rate, blood pressure and brief neurological exam. The Mini International Neuropsychiatric Interview version 5 (MINI-5) was performed by an experienced psychiatrist to assess mental health. Additional exclusion criteria included a diagnosis of a psychiatric (ICD-10 or DSM-IV Axis I) requiring current psychological or pharmacological treatment, neurological or endocrine illnesses, a history of drug or alcohol dependence, having a Body Mass Index below 19 or above 24 and being a non-native English speaker. Participants with nicotine dependence were not excluded. Ethnicity was not recorded.

Assessments

Empathic Stories Task

This task aimed to assess emotional empathy by assessing participants' emotional reactions in response to stories with different emotional themes. Participants were given 6 different stories to read on each visit day. There were 2 "happy" themed stories, 2 "angry" themed stories, 2 "sad" themed stories. Stories were a one paragraph description of a situation. For

example, a “happy” story described a family reunion at an airport, a “sad” story described a family being in a car accident where one of the children died, an “angry” story described someone missing a flight due to train cancellations.

Participants were asked to rate how “good” (any positive emotion– for example happy, pleased, hopeful, cheerful) or “bad” (any negative emotion– for example unhappy, scared, angry, melancholic) the stories made them feel. These ratings relate to “implicit” emotional empathy – that is, amount of emotional arousal in response to stimuli (Mehrabian and Epstein, 1972; Dziobek et al., 2008). Participants were asked to rate their personal, “gut-feeling” reaction by picking a self-assessment manikin (SAM) best corresponding to the positivity or negativity of their emotion on a scale of 1 (most positive) to 9 (most negative). Self-assessment manikins have been found to be a good way of assessing emotional response (Bradley and Lang, 1994). This score of 1-9 was analysed as the dependent variable.

See Figure S1 for an example of the self-assessment Manikin scale used in the study.

Trust investment (Berg et al., 1995)

Participants made a total of 20 decisions. Participants were told they had £500, which they could choose to invest in 20 different entrepreneurs, played by a computer. They were told all entrepreneurs were highly skilled, but not all were trustworthy. Participants were told they might be able to triple their original investment if they chose wisely, or that they may lose all their money if not. Participants could choose to not send any money and therefore keep their £500. They were shown the face of the individual running the business and asked to choose an amount that they wished to invest. Faces the participants were shown were computer generated with neutral expressions. Participants were told their goal was to maximise the amount of money they would keep. Participants were not given any feedback

on the outcome of each investment until the end of the task. Participants were told they would be paid the amount of money remaining after one randomly chosen trial, divided by 100. This was added to the standard reimbursement they received for taking part. The 'entrepreneurs' responses were pre-determined.

Cooperative behaviour games:

Participants were told they were playing these games with all the other participants in the study. These tasks were done on paper. The tasks were organised as 'one-shot' games. Participants were told that one of their responses would be chosen at random and this would be paid to them in addition to their standard reimbursement for taking part.

Dictator Game (Hoffman et al., 1996)**:** Every participant played the role of the 'dictator' at both of their sessions, with another study participant (unknown to them) being affected by their decision. Participants completed one trial at each session. Both parties would receive the amount that the 'dictator' chose.

Ultimatum Game (Thaler, 1988; Guth and Tietz, 1990): For both roles, participants were told that their response to this game from one of their sessions would be randomly paired with another participants' and they would both receive the result of that decision. Every participant completed one trial as the proposer and one trial as the decider.

The following tasks have been shown to be sensitive to recreational MDMA: Trustworthy Face Rating, Dictator Game, Ultimatum Game (Stewart et al., 2014) and MDMA: Ultimatum Game (Gabay et al., 2019).

A task similar to our Trust Investment Task (Trust Game) used by Kosfeld et al. (2005) was sensitive to the effects of oxytocin. Their study did not include pictures of faces.

Participants were not trained on the tasks described in this report.

Results

Demographics

Table S1 reports participants' lifetime drug history. Data were missing for four participants, so we analysed 21 participants' data.

Task Results

See Table S2 for full task results.

Table S3 lists full statistics for all Mood & Symptom VAS analyses.

Subacute Mood & Symptom VAS and BDI

Table S4 lists all subacute Mood & Symptom VAS and questionnaire analyses.

Tasks results

Empathic Stories Task

The significant main effect of story Emotion reflected that ‘Happy’ stories led participants to feel more positive than ‘Angry’ stories (t_{20}=-9.011, p<0.001, mean difference=-3.262, 95% CI: -4.207 to -2.317), and ‘Sad’ stories led to more negative feelings than both ‘Happy’ (t_{20}=10.174, p<0.001, mean difference=4.619, 95% CI: 3.432 to 5.806) and ‘Angry’ stories (t_{20}=5.702, p<0.001, mean difference=1.357, 95% CI: 0.735 to 1.979).

Trustworthiness ratings of faces

14 received MDMA at first visit, placebo at second visit; 10 received placebo at first visit, MDMA at second visit. After adding drug order, a significant Drug by Face Gender by Order interaction emerged ($F_{1,22}$=4.797, p=0.039, η_p^2=0.179). Female faces were rated as more trustworthy than male faces under MDMA (t_{13}=2.21, p=0.025, 95% CI: 0.041 to 0.547), but only for participants who received MDMA at their first visit. See Figure S2 for representation of this effect.

Trust Investment Task

13 received MDMA at first visit, placebo at second visit; eight received placebo at first visit, MDMA at second visit. After adding Order as an additional factor, we found a significant interaction between Drug and Order ($F_{1,19}$=11.923, p=0.003, η_p^2=0.386). Exploration of the drug by order interaction showed that when participants received MDMA first they invested more money under the placebo condition than the MDMA condition, (t_{12}= 3.73, p=0.001, mean difference=1594.538, 95% CI: 700.225 to 2488.851). When participants received

placebo first, there was no significant difference in the amount of money invested between conditions (t_7=-1.461, p=0.160, mean difference=-795.875, 95% CI: -1935.905 to 344.155). We also assessed an effect of Session Day. There was no interaction between Session Day and Order ($F_{1,19}$=1.331, p=0.263, η_p^2=0.065), however there was a main effect of Session Day ($F_{1,19}$=11.923, p=0.003, η_p^2=0.386) reflecting a higher amount invested at the second session than the first session (t_{20}=3.453, p=0.003, mean difference=1195.207, 95% CI: 470.730 to 1919.683).

Correlations

We found no significant correlations between plasma MDMA levels and task performance. We note a negative correlation between emotional ratings on "Sad" stories and plasma MDMA levels at two hours, which was significant at trend level (r=-0.550, p=0.015). This correlation reflects a less negative emotional response to stories with a "Sad" valence as plasma MDMA levels increased. Please see Table S5-S8 for full results.

Supplementary discussion

We found a significant order effect on our Trust Investment task, which could suggest that the effects of MDMA carryover to later testing sessions. This may have relevance for the psychotherapeutic application of MDMA, whereby drug-assisted sessions are integrated over subsequent drug-free psychotherapy sessions (Mithoefer et al., 2016). However, in a separate analysis, there was a significant effect of Session Day: participants were more trusting with their financial allocations at their second session. This offers an alternative explanation to our Drug by Order interaction; participants may be simply more willing to invest on their second visit. This may be related to familiarity, which is recognised as an important component of economic decision making and may also affect trust (Cao et al., 2011). However, we cannot determine what caused this effect from our data- a study where drug order is experimentally manipulated would be necessary. Particularly as no previous laboratory MDMA studies have found order effects on these tasks, any interpretation of these results must remain tentative.

In the Public Good Game, our participants donated close to the maximum under placebo, and over £1 more than the control participants in the Stewart et al (2014) study. Our ability to detect an effect of MDMA may have therefore been limited. It is interesting to explore our results for the Ultimatum Game in more detail. In the 'Decider' condition, our participants were willing to accept 37% of the total stake under placebo, 34% of the total stake under MDMA. Brandts and Charness (2011) argue that the method we used elicits less punishment than the 'direct-response' method used by Gabay et al. (2018). Our results would align with this, given that offers of below 40% of the total stake are not considered 'fair' (Gabay et al.,

2014). Interestingly, the participants in Gabay et al. (2018) made lower proposals (48.2% of the total stake in the placebo condition in vs 62.5% of the total stake in our placebo condition; 55.7% in the MDMA condition vs 69.5% in our MDMA condition). Again, this may have limited our ability to detect an effect of MDMA increasing generosity or reducing punishment.

We found a trend for a negative correlation between plasma MDMA levels at two hours and empathic response ratings on the "Sad" stories. Given that we found no effect of drug on this task, we cannot draw any firm conclusions from this trend result. However, it is interesting to see a correlation between a biological measure and a psychological effect that has been previously noted – that MDMA reduces the impact of negative stimuli (Carhart-Harris et al., 2014; Frye et al., 2014). This would merit further study.

Further limitations

We used a novel task for our measure of empathy, as opposed to a more validated method such as the Multifaceted Empathy Test. Thus, our task may have lacked sensitivity to detect an effect of MDMA. As we had limited power, we were also not able to assess the impact of sex differences, which have been noted as relevant in MDMA research (Allott and Redman, 2007).

Table S1 Recent and lifetime drug history

	Min	Max	Median	Interquartile range (IQR)	N
**Recent drug use**					
Alcohol (weekly units)	0	35	13	3-19	21
Cigarettes (daily number)	0	3	0	0-1	21
Cigarettes (days since last use)	0.75	2920	2	551.83	9
Cannabis (days since last use)	2	8760	90	1455	19
**Lifetime drug use (number of times)**					
MDMA	1	200	10	3-45	21
Cannabis	0	1000	150	20-500	21
LSD	0	500	1	0-5.5	21
Psilocybin	0	100	1	0-10	21
Ketamine	0	200	1	0-17.5	21
Mephedrone	0	30	0	0-4	21
Amphetamine	0	150	0	0-35	21
Cocaine	0	200	4	0-20	21

Table S2 Task results with mean values (standard deviation) reported.

**Empathic Stories Task emotional response rating**	**MDMA**	**Placebo**	**JZS Bayes Factor**
“Happy” story	2.786 (1.347)	2.857 (1.296)	5.821
“Sad” story	7.476 (1.308)	7.405 (1.633)	5.898
“Angry” story	5.786 (1.210)	6.381 (0.773)	1.101
**Trustworthy Face Rating**			
Female Face	4.071 (0.450)	3.923 (0.525)	1.837
Male Face	3.927 (0.585)	3.890 (0.690)	5.768
**Trust Investment Task (amount invested out of £10000)**			
	4740.095 (2413.100)	5424.000 (2429.796)	1.780

	Baseline 0 hour	2 hours post	4 hours post	Baseline 0 hour	2 hours post	4 hours post	Drug	Time	Drug x Time	Drug x Time x Order	Time 0 (MDMA vs placebo)	Time 2 (MDMA vs placebo)	Time 4 (MDMA vs placebo)	MDMA (Time 0 vs 2), Time 0 vs 4	Placebo (Time 0 vs 2, time 0 vs 4)
**General drug effects**															
Euphoria	1.250 (2.023)	6.750 (2.653)	2.200 (2.353)	1.550 (2.481)	1.050 (1.820)	0.500 (1.192)	**$F_{1,19}$=39.594 p<0.001 η_p^2 =0.676**	**$F_{2,38}$=29.128 p<0.001 η_p^2 =0.605**	**$F_{2,38}$=44.519 p<0.001 η_p^2 =0.701**	$F_{2,36}$=1.012 p=0.373 η_p^2 =0.053	t_{19}=-0.645 p=0.527	**t_{19}=9.194 p<0.001**	**t_{19}=3.448 p=0.003**	**t_{19}=-8.197 p<0.001; t_{19}=-1.900 p=0.218**	t_{19}=1.190 p=0.745; t_{19}=2.303 p=0.098
Drug effect	0.250 (1.118)	6.500 (2.838)	3.100 (3.059)	0.250 (1.118)	0.600 (1.698)	0.400 (1.273)	**$F_{1,19}$=57.031 p<0.001 η_p^2 =0.750**	**$F_{2,38}$=43.406 p<0.001 η_p^2 =0.696**	**$F_{2,38}$=43.125 p<0.001 η_p^2 =0.694**	$F_{2,36}$=1.431 p=0.252 η_p^2 =0.074	Identical ratings at time 0 for MDMA/placebo	**t_{19}=9.035 p<0.001**	**t_{19}=4.212 p<0.001**	**t_{19}=-9.585 p<0.001; t_{19}=-4.222, p=0.001**	t_{19}=-1.159 p=0.780; t_{19}=-1.000 p=0.990
Jaw clenching	0.750 (1.803)	4.750 (3.291)	2.950 (3.137)	1.550 (2.523)	1.200 (2.191)	0.750 (1.410)	**$F_{1,19}$=23.339 p<0.001 η_p^2 =0.551**	**$F_{2,38}$=17.933 p<0.001 η_p^2 =0.486**	**$F_{2,38}$=14.812 p<0.001 η_p^2 =0.438**	$F_{2,36}$=1.821 p=0.176 η_p^2 =0.092	t_{19}=-1.670, p=0.111	**t_{19}=5.007 p<0.001**	**t_{19}=4.104, p=0.001**	**t_{19}=-5.540, p<0.001; t_{19}=-3.443, p=0.008**	t_{19}=1.129 p=0.819; t_{19}=1.848, p=0.241
Energy	6.300 (2.130)	6.950 (2.964)	6.300 (2.386)	6.350 (1.814)	5.900 (2.049)	5.600 (2.458)	$F_{1,19}$=3.228 p=0.088 η_p^2 =0.145	$F_{2,38}$-0.601 p=0.553 η_p^2 =0.031	$F_{2,38}$=.677 p=0.514 η_p^2 =0.034	$F_{2,36}$=1.262 p=0.295 η_p^2 =0.066					
**Prosocial effects**															
Trusting of others	2.000 (1.947)	2.150 (2.346)	1.850 (1.954)	1.550 (1.761)	1.750 (1.773)	1.750 (1.916)	$F_{1,19}$=0.679 p=0.420 η_p^2=0.035	$F_{2,38}$=0.266 p=0.768 η_p^2=0.014	$F_{2,38}$=0.211 p=0.811 η_p^2=0.011	$F_{2,36}$= 0.344 p=0.711 η_p^2=0.019					
Empathy	5.53 (2.195)	6.630 (2.733)	5.950 (2.297)	5.050 (2.121)	5.110 (2.447)	5.00 (2.333)	$F_{1,18}$=10.073 p= 0.005 η_p^2=0.359	$F_{1.410,25.384}$=2.202 p=0.143 η_p^2=0.109	$F_{2,36}$ =1.792 p=0.181 η_p^2=0.091	$F_{1.799,30.590}$=0.213 p=0.787 η_p^2=0.012					
Friendly	1.700 (2.273)	1.050 (2.188)	1.500 (2.626)	1.750 (2.197)	2.050 (2.417)	1.750 (2.173)	$F_{1,19}$=0.944 p=0.343 η_p^2=0.047	$F_{2,38}$=0.260 p=0.752 η_p^2=0.014	$F_{1.349,25.629}$=2.073 p=0.158 η_p^2=0.098	$F_{1.375,24.756}$= 1.238 p=0.293 η_p^2=0.064					
Closeness to others	4.900 (2.049)	6.400 (2.909)	5.350 (2.601)	4.250 (2.447)	4.600 (2.722)	5.300 (2.227)	$F_{1,19}$=6.013 p=0.024 η_p^2=0.240	$F_{2,38}$=3.719 p=0.033 η_p^2=0.164	**$F_{2,38}$=8.010 p=0.001 η_p^2=0.297**	$F_{2,36}$=2.056 p=0.143 η_p^2 =0.103	**t_{19}=2.218 p=0.039**	**t_{19}=3.636 p=0.002**	t_{19}=0.108 p=0.915	**t_{19}=-2.941 p=0.025;** t_{19}=-1.014 p=0.971	t_{19}=-0.837 p=1.00; t_{19}=-2.365 p=0.087
Amicable	2.400 (2.798)	1.800 (2.949)	1.900 (2.532)	1.800 (2.093)	1.900 (2.245)	1.955 (2.139)	$F_{1,19}$=.167 p=0.687 η_p^2=0.009	$F_{2,38}$=0.387 p=0.682 η_p^2=0.020	$F_{1.338,25.414}$=0.666 p=0.464 η_p^2=0.034	$F_{1.324,23.829}$=0.382 p=0.601 η_p^2=0.021					
Want to be with others	5.550 (1.791)	7.050 (2.328)	6.450 (2.012)	5.550 (1.820)	5.600 (1.536)	5.40 (1.984)	$F_{1,19}$=4.046 p=0.059 η_p^2 =0.176	$F_{2,38}$=3.460 p=0.042 η_p^2 =0.154	$F_{1.310,24.894}$=2.660 p=0.107 η_p^2 =0.123	$F_{1.278,23.003}$ =0.291 p=0.651 η_p^2=0.016					
Compassionate	3.320 (2.583)	2.160 (2.968)	2.740 (2.746)	3.420 (2.610)	3.260 (2.579)	3.840 (2.522)	$F_{1,18}$=8.041 p=0.011 η_p^2 =0.309	$F_{2,38}$=3.767 p=0.033 η_p^2 =0.173	$F_{2,36}$=2.295 p=0.115 η_p^2 =0.113	$F_{2,34}$=0.624, p=0.542, η_p^2=0.035					

*Table S3 Subjective Effects- Acute – mean (standard deviation) self-ratings VAS 0-10 of MDMA and placebo groups at pre-drug 0 hour, 2 hour post-drug, 4 hour post-drug and test statistics (F and t) for RM-ANOVA and post-hoc pairwise comparisons (Bonferroni-corrected). When required, F tests were Greenhouse-Geisser corrected. The α for the F test was Bonferroni corrected by dividing by 11 to give α=0.0045. Significant results are highlighted in **bold**.*

	MDMA		Placebo		F (α =0.01)				t	JZS Bayes Factor
	**Baseline 0 hour**	**3 days post-drug**	**Baseline 0 hour**	**3 days post-drug**	**Drug**	**Day**	**Drug x Day**	**Drug x Day x Order**	**Baseline vs Day 3**	
Happy – sad (n=17)	2.240 (2.251)	2.240 (1.522)	2.060 (1.784)	2.120 (2.088)	$F_{1,16}$=0.282 p=0.603 η_p^2 =0.017	$F_{1,16}$=0.011 p=0.919 η_p^2 =0.001	$F_{1,16}$=0.008 p=0.930 η_p^2 =0.001	$F_{1,15}$=0.011 p=0.919 η_p^2 =0.001		5.443
Calm – anxious (n=17)	3.000 (2.208)	1.940 (1.435)	2.710 (1.993)	1.880 (1.900)	$F_{1,16}$=0.269 p=0.611 η_p^2 =0.017	**$F_{1,16}$=11.506 p=0.004 η_p^2 =0.418**	$F_{1,16}$=0.119 p=0.735 η_p^2 =0.007	$F_{1,15}$=1.031 p=0.326 η_p^2 =0.064	**t_{16}=3.397 p=0.004**	1.110
Trusting of others - Distrusting of others (n=16)	1.940 (2.048)	2.130 (1.746)	1.880 (1.928)	2.370 (1.668)	$F_{1,15}$=0.064 p=0.804 η_p^2 =0.004	$F_{1,15}$=1.076 p=0.316 η_p^2 =0.067	$F_{1,15}$=0.311 p=0.585 η_p^2 =0.020	$F_{1,14}$=0.063, p=0.806, η_p^2 =0.004		4.992
Want to be alone - Want to be with others (n=16)	5.690 (1.493)	5.810 (1.721)	5.250 (0.775)	5.750 (1.238)	$F_{1,15}$=0.732 p=0.406 η_p^2 =0.047	$F_{1,15}$=0.940 p=0.348 η_p^2 =0.059	$F_{1,15}$=0.368 p=0.553 η_p^2 =0.024	$F_{1,14}$=0.776 p=0.393 η_p^2 =0.052		5.161
No empathy – extreme empathy (n=16)	5.500 (1.592)	5.440 (1.931)	5.310 (1.662)	5.310 (1.991)	$F_{1,15}$=0.701 p=0.416 η_p^2 =0.045	$F_{1,15}$=0.007 p=0.935 η_p^2 =0.000	$F_{1,15}$=0.011, p=0.917 η_p^2 =0.001	$F_{1,14}$=5.939 p=0.029 η_p^2 =0.298		5.241
BDI (n=15)	2.130 (3.114)	1.270 (2.344)	1.330 (1.877)	1.400 (2.473)	$F_{1,14}$=0.459 p=0.509, η_p^2 =0.032	$F_{1,14}$=0.599, p=0.452, η_p^2 =0.041	$F_{1,14}$=0.876, p=0.365, η_p^2 =0.059	$F_{1,13}$=0.100 p=0.756 η_p^2 =0.008		3.096

Table S4 - Subacute effects, comparison of pre-drug baseline on acute days vs 3 days post drug. JZS Bayes Factor are calculated from t-statistic comparing pre-MDMA and 3 days post-MDMA results. The α for the F test was Bonferroni corrected to 0.01.

Table S5 Correlations between plasma MDMA levels and Task results. α =0.005

		**Trust Investment Task**	**Public Good Game**	**Dictator Game**	**Ultimatum Game**		**Trustworthiness rating**		**Empathic stories task**		
					Proposer	Decider	Male faces	Female faces	Happy	Sad	Angry
**Plasma MDMA levels (2 hours post-drug)**	**Pearson *r***	-0.116	-0.305	-0.015	0.014	0.226	-0.339	-0.381	0.280	-0.550	-0.221
	p	0.616	0.190	0.950	0.953	0.339	0.123	0.080	0.245	0.015	0.364

Table S6 Correlations between plasma plasma MDMA levels and Mood & Symptom VAS results. α =0.01

		**Euphoria**	**Jaw clenching**	**Feel drug effect**	**Closeness to others**
**Plasma MDMA levels (2 hours post-drug)**	**Pearson *r***	-0.161	0.334	-0.029	-0.109
	p	0.497	0.150	0.902	0.646

Table S7 Correlations between VAS measured 'Trust' at 2 hours post-drug and task-measured trust. α =0.008

**Trusting VAS**	**Trustworthiness rating**		**Trust investment**
	**Male faces**	**Female faces**	
**Pearson *r***	-0.478	-0.322	-0.029
p	0.028	0.154	0.904

Table S8 Correlations between VAS measured 'Empathy' at 2 hours post-drug and task-measured empathy. α =0.008

**Empathy VAS**	**Empathic stories task**		
	**Happy**	**Angry**	**Sad**
**Pearson *r***	0.255	0.020	0.162
p	0.264	0.932	0.483

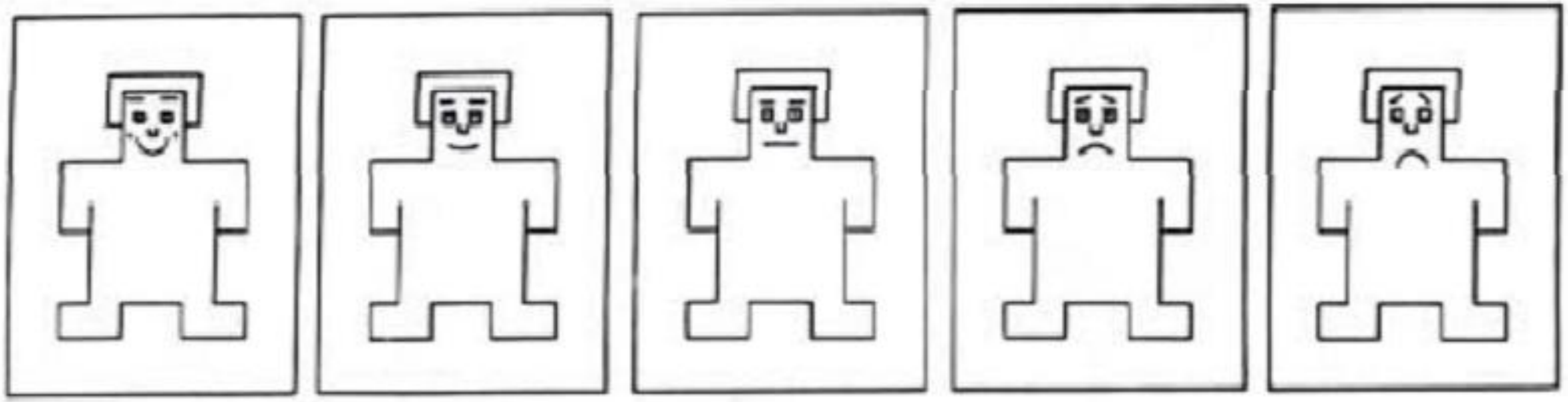

Figure S1 Self-assessment Manikin - participants could choose 1 - 9, including space between specific manikins, to correspond to the positivity or negativity of the emotion they felt in response to the task. Figure adapted from Bradley & Lang 1994.

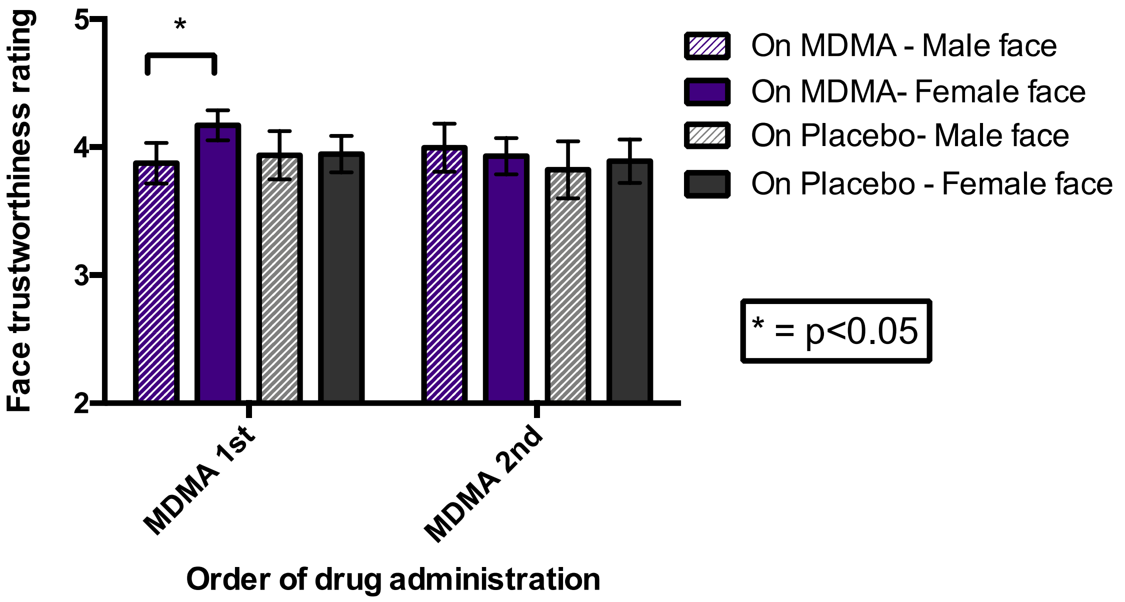

*Figure S1 Face trustworthiness ratings are affected by session order. Participants rate female faces highest post-MDMA, but only when this is their first session within the study. *=p<0.05*

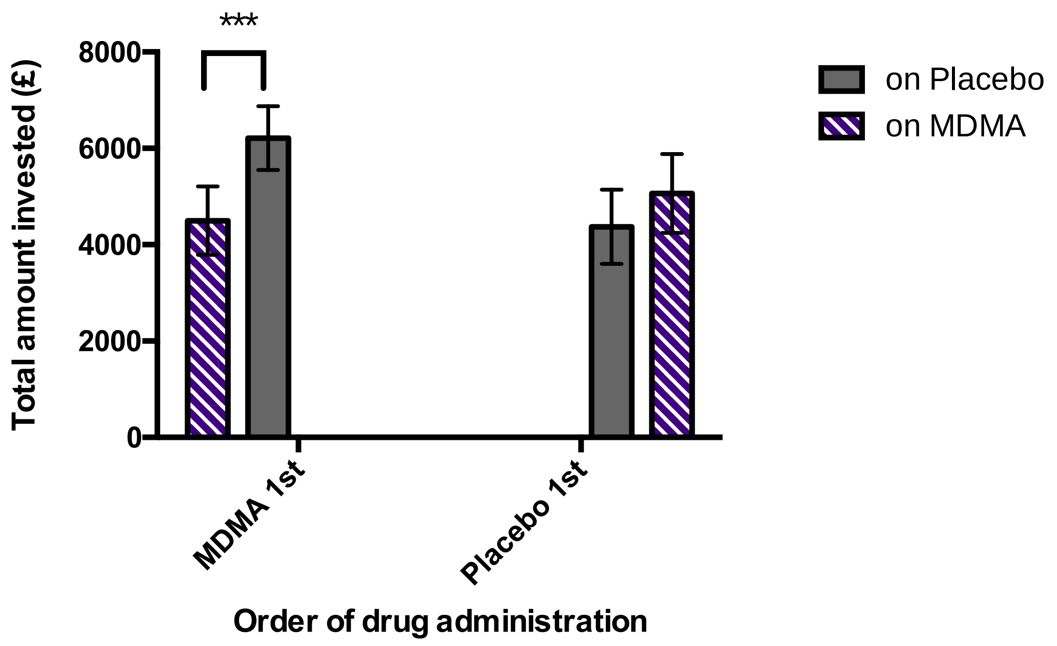

Figure S3. Trust Investment (a trust-related behaviour) is affected by drug order, with participants investing more money post-placebo when they had received MDMA at their previous session.

*This can alternatively be explained by an overall greater investment on the second visit compared to the first visit. ***= p=0.001*

References

Allott K and Redman J. (2007) Are there sex differences associated with the effects of ecstasy/3,4-methylenedioxymethamphetamine (MDMA)? *Neuroscience & Biobehavioral Reviews* 31: 327-347.

Berg J, Dickhaut J and McCabe K. (1995) Trust, Reciprocity, and Social History. *Games and Economic Behavior* 10: 122-142.

Bradley MM and Lang PJ. (1994) Measuring emotion: the Self-Assessment Manikin and the Semantic Differential. *J Behav Ther Exp Psychiatry* 25: 49-59.

Brandts J and Charness G. (2011) The strategy versus the direct-response method: a first survey of experimental comparisons. *Experimental Economics* 14: 375-398.

Cao H, Han B, Hirshleifer D, et al. (2011) Fear of the Unknown: Familiarity and Economic Decisions. *Review of Finance* 15: 173-206.

Carhart-Harris RL, Wall MB, Erritzoe D, et al. (2014) The effect of acutely administered MDMA on subjective and BOLD-fMRI responses to favourite and worst autobiographical memories. *The International Journal of Neuropsychopharmacology* 17: 527-540.

Dziobek I, Rogers K, Fleck S, et al. (2008) Dissociation of cognitive and emotional empathy in adults with Asperger syndrome using the Multifaceted Empathy Test (MET). *J Autism Dev Disord* 38: 464-473.

Frye CG, Wardle MC, Norman GJ, et al. (2014) MDMA decreases the effects of simulated social rejection.

Gabay AS, Carhart-Harris RL, Mazibuko N, et al. (2018) Psilocybin and MDMA reduce costly punishment in the Ultimatum Game. *Scientific Reports* 8: 8236-8236.

Gabay AS, Kempton MJ, Gilleen J, et al. (2019) MDMA Increases Cooperation and Recruitment of Social Brain Areas When Playing Trustworthy Players in an Iterated Prisoner's Dilemma. *The Journal of Neuroscience* 39: 307-320.

Gabay AS, Radua J, Kempton MJ, et al. (2014) The Ultimatum Game and the brain: a meta-analysis of neuroimaging studies. *Neurosci Biobehav Rev* 47: 549-558.

Guth W and Tietz R. (1990) *Ultimatum Bargaining Power*: North-Holland Pub. Co.

Hoffman E, McCabe K, Smith VL, et al. (1996) Social Distance and Other-Regarding Behavior in Dictator Games. *American Economic Review* 86: 653-660.

Kosfeld M, Heinrichs M, Zak PJ, et al. (2005) Oxytocin increases trust in humans. *Nature* 435: 673-676.

Mehrabian A and Epstein N. (1972) A measure of emotional empathy. *J Pers* 40: 525-543.

Mithoefer MC, Grob CS and Brewerton TD. (2016) Novel psychopharmacological therapies for psychiatric disorders: psilocybin and MDMA. *The Lancet Psychiatry* 3: 481-488.

Stewart LH, Ferguson B, Morgan CJA, et al. (2014) Effects of ecstasy on cooperative behaviour and perception of trustworthiness: A naturalistic study. *Journal of Psychopharmacology* 28: 1001-1008.

Thaler RH. (1988) Anomalies: The Ultimatum Game. *Journal of Economic Perspectives* 2: 195-206.
